# Assessment in mathematics: a study on teachers’ practices in times of pandemic

**DOI:** 10.1007/s11858-022-01395-x

**Published:** 2022-07-20

**Authors:** Annalisa Cusi, Florian Schacht, Gilles Aldon, Osama Swidan

**Affiliations:** 1 Sapienza Università di Roma, Rome, Italy; 2 Universität Duisburg-Essen, Essen, Germany; 3 IFÉ-ENS de Lyon, University of Lyon, Lyon, France; 4 Ben Gurion University of the Negev, Beer-Sheva, Israel

**Keywords:** COVID-19 pandemic, Distance teaching, Formative assessment, Summative assessment, Meta-didactical transposition, Praxeologies

## Abstract

Lockdowns imposed by many countries on their populations at the beginning of the COVID-19 crisis forced teachers to adapt quickly and without adequate preparation to distance teaching. In this paper, we focus on one of the most formidable challenges that teachers faced during the lockdowns and even in the post-lockdown emergency period, namely, developing assessment that maintains the pedagogical continuity that educational institutions typically require. Based on the results of a previous study, focused on the analysis of answers to an open-ended questionnaire administered to a population of 700 teachers from France, Germany, Israel and Italy, a semi-structured interview series was designed and implemented by the authors of this paper with a small group of teachers. The transcripts of these interviews were analysed according to the interpretative phenomenological analysis methodology, with the aim of investigating teachers’ own perspectives on the following: (a) the difficulties with which they had to contend, with respect to the question of assessment; (b) the techniques adopted to deal with these difficulties; and (c) the ways in which the lockdown experience could affect the future evolution of teachers’ assessment practices. This analysis supported us in formulating hypotheses concerning the possible long-term effects of lockdown on modes of assessment in mathematics.

## Introduction and literature review

Due to the dramatic change in the school structure and the widespread shift to distance teaching caused by the unexpected COVID-19 emergency, systemic societal and educational problems have become more visible to a wider community, boosting researchers “to think about the potential of a new normal” (Bakker et al., 2021, p. 5).

Therefore, a rich debate has been triggered to share reflections about the main consequences that this emergency could have for mathematics education. This debate enabled researchers to highlight big challenges that this situation has brought or amplified, such as the risk of falling back to pedagogies more focused on the transmission of knowledge and the creation of new boundaries for communication (Bakker & Wagner, 2020) or the contrast between the potentialities provided by digital resources in fostering the creation of effective interactive environments for teachers and students and the risk that the pandemic situation could have amplified the social gap that exists in the world (Engelbrecht et al., 2020). These considerations led to reflections on the role of the COVID-19 crisis in having pushed forward the agenda of the digital technology trend in mathematics education (Borba, 2021) and on the ways in which mathematics education could contribute in providing citizens with the necessary tools to face global crises like the one in which we all have been involved (Krause et al., 2021). Borba (2021), in particular, suggested reflecting on the role of crisis as a “chance for change” (p. 389). In tune with this idea, different studies have been developed to reflect on how the pandemic era has affected the teaching–learning of mathematics and on the possible changes in the future of mathematics education. Some of these studies, for example, investigated the ways in which the emergency situation has inspired teachers to find solutions to problems they had not encountered before (Flores & Swennen, 2020), such as completely reconstructing the didactic system (Albano et al., 2021) or developing their professional growth in new and unprecedented environments (Huang et al., 2022).

Assessment has been identified as one of the key topics and issues for future research, highlighted within this rich debate. In relation to this issue, Bakker et al. (2021), in particular, stressed the need to reflect on facing the challenge of how to “successfully assess what we value rather than merely assessing what is relatively easy to assess” (p. 18).

The role of assessment as a challenging issue in mathematics education during the COVID-19 period has been highlighted in different research studies, which focused on the following: (a) the design of assessment items to be used in distance teaching (Fitzmaurice & Ní Fhloinn, 2021; Frost et al., 2021); (b) the use of specific technological tools in assessing students’ learning (McLaughlin et al., 2021); and (c) the effects of the pandemic on students’ skills (Pócsová et al., 2021).

Some studies focused also on teachers’ perspectives on the issue of carrying out assessment in times of pandemic. Nilsberth et al. (2021), for example, highlighted the centrality of the discourse on assessment for teachers, observing that assessment represented one of the discursive frames that teachers relied on when they developed the pedagogical considerations that guided their decisions during the COVID-19 crisis. Assessment formats used by teachers and their opportunities and limitations were one of the foci of the quantitative study developed by Drijvers et al. (2021), who located “the opportunities for formative and summative assessment” among the four perspectives that support the description of teachers’ preparation and delivery of teaching practices at distance. In particular, they highlighted that formative assessment represents an important issue in distance mathematics education, due to teachers’ limited confidence with respect to the use of digital means to provide formative feedback to students. Similarly, Aldon et al. (2021), through their qualitative analysis of 700 mathematics teachers’ answers to an open-ended questionnaire, showed that assessment represented a huge challenge for teachers during the lockdown period, which forced most of them to adopt a formative assessment perspective. Formative assessment represented a crucial issue also for university lecturers, as highlighted by Fitzmaurice and Ní Fhloinn (2021), who noticed that, during the lockdown period, a broader range of assessment methods were embraced by mathematics lecturers.

We think that, in order to shed light on how the pandemic affected teachers’ assessment practices, the description of teachers’ ways of facing the challenge of carrying out assessment during the COVID-19 emergency needs to be integrated with their interpretations of the complex phenomena in which they have been involved. In line with this idea, the study documented in this paper, which builds upon the results of a previous study aimed at exploring teachers’ perspectives on how the lockdown period affected their practices (Aldon et al., 2021), is focused on data collected through a semi-structured interview series with a group of teachers from four countries (France, Germany, Israel and Italy). By means of a fine-grained analysis of teachers’ reflections on the evolution of their assessment practices during both the lockdown and the post-lockdown emergency period, in this study we addressed the following aims: (a) identify the main challenges that mathematics teachers faced during the pandemic in relation to assessment practices; (b) discuss how teachers dealt with such challenges; and (c) make hypotheses on how the pandemic may affect the evolution of teachers’ assessment practices, by focusing on how teachers foresee this evolution.

The investigation of these three issues carries important theoretical and pedagogical implications. From the theoretical point of view, in this study we aim to shed new light on the factors influencing teachers’ choices in relation to assessment practices and on how teachers interpret and justify their choices. From the pedagogical point of view, this study supports the formulation of hypotheses concerning the possible long-term effects of lockdown on modes of assessment in mathematics, providing ideas that could support educators and policymakers in the design of teachers’ professional development programmes.

## Research framework and research questions

The results of the studies on assessment during the COVID-19 emergency period, documented in the previous section, are in tune with research on assessment. Going from the paradigm of pragmatic intuition (Eşi, 2014) to the paradigm of assessment as learning (Black & Wiliam, 2009), assessment is, in fact, always a fundamental concern for teachers. What distinguishes formative assessment from other kinds of assessment is its use in the process of learning: it focuses on data about students’ performance, collected during teaching and learning activities, with the aim of making “decisions about the next steps in instruction that are likely to be better, or better founded, than the decisions they would have taken in the absence of the evidence that was elicited.” (Black & Wiliam, 2009, p. 7). Wiliam and Thompson (2007) identified five key strategies aimed at fostering formative assessment processes: (1) clarifying and sharing learning intentions and criteria for success; (2) engineering effective classroom discussions and other learning tasks that elicit evidence of student understanding; (3) providing feedback that moves learners forward; (4) activating students as instructional resources for one another; and (5) activating students as the owners of their own learning.

Digital technologies could play a key role in supporting the activation of these strategies. In particular, by means of digital technologies, fundamental dimensions of formative assessment could be supported, in the following ways: (a) continuity, by enabling teachers to keep track of students’ learning (Roschelle & Pea, 2002); (b) regulation, by providing students with immediate feedback and encouraging them to monitor their own progress (Gikandi et al., 2011); (c) collaboration, by providing opportunities for students to peer- and self-assess their work (Clark-Wilson, 2010); and (d) participation, by encouraging the students’ dynamic engagement in conceptual activities (Ares, 2008).

The key role played by digital technologies becomes particularly relevant when assessment processes have to be developed at a distance, as during the lockdown period. As previously stated, in this paper we focus on teachers’ perspectives on the effects of the COVID-19 experience on their assessment practices. Therefore, we searched for a theoretical lens useful to support both the description of what teachers do and implement in their professional context, and the analysis of the ways in which they justify their actions and choices, by referring to the knowledge involved.

For this reason, we referred to the Meta-Didactical Transposition model (MDT) (Arzarello et al., 2014; Cusi et al., 2022 (in print)) as a fundamental lens through which we analysed teachers’ reflections on the lockdown and post-lockdown teaching experience. This model, in fact, is effective both in describing the activities conducted by teachers during their teaching processes, and in investigating the reasons that guided their choices.

The MDT is based on the Anthropological Theory of Didactics (Chevallard, 1985), which interprets mathematics teaching as an ordinary human activity, carried out within institutions. According to this theory, mathematics teaching is characterized through the notion of praxeology, structured on two levels (García et al., 2006), namely, the *praxis* (know how) and the *logos* (know why). The *task* to be faced, and the *techniques* adopted for facing the task are components of the praxis level, while the discourse developed to justify and validate the techniques (*technology*) and the elements that provide the basis and support for this technological discourse (*theory*) constitute the logos level. In the following, we refer to the logos level using the term *justifying discourses* (Arzarello et al., 2014).

In work by Arzarello et al. (2014), the term *meta-didactical praxeology* was introduced to denote the specific praxeologies that emerge from teachers’ (and researchers’) reflections on the didactical praxeologies, which refer to the knowledge to be taught and to the techniques recognized and justified within specific institutions. In line with Aldon et al. (2021), in this paper we refer to the notion of meta-didactical praxeologies to investigate how teachers managed their assessment practices, both during the lockdowns and in the post-lockdown emergency period, since our focus is not only on teachers’ descriptions of the ways in which they adapted their usual didactical praxeologies, but also on the justifications they provide about the choices they made and on their reflections about this experience, which positioned their discourses at a meta-level.

Praxeologies continuously evolve, due to the different experiences that teachers live and to the corresponding reflections that these experiences trigger, within different institutional contexts. Another key theoretical lens offered by MDT is, therefore, the notion of *internalization*, introduced by Arzarello et al. (2014) to indicate the phenomenon at the base of this evolution. Through the internalization process, new components are integrated within existing (didactical or meta-didactical) praxeologies. This process is triggered by teachers’ experiences (participating in professional development programmes or collaborative research projects, but also facing specific challenges during critical periods in their professional lives, such as the COVID-19 crisis) and by the reflections they develop from these experiences.

Aldon et al. (2021) showed that two main praxeologies related to the task of managing distance teaching to develop assessment practices emerged from teachers’ discourses about the ways in which they reacted to the challenges faced during the lockdown period. Although some teachers were shown to have adopted a summative perspective on assessment (first praxeology), most of the teachers who participated in the study reflected on their assessment practices during the lockdown period focusing on strategies that are clearly connected to those presented by Wiliam and Thompson (2007), highlighting the prevalence of a formative assessment perspective (second praxeology). These results motivated us to continue investigating teachers’ perspectives concerning assessment during the COVID-19 crisis, with the aim of highlighting, in tune with Drjivers et al.’s (2021) recommendations for future research, whether or not our findings would be confirmed after some time had elapsed, as teachers familiarised themselves with the emergency situation. In particular, in this paper we present the results of our investigation of teachers’ perspectives on the evolution, during the lockdown and post-lockdown experiences, of their praxeologies related to the task of assessing their students, by focusing on the following research questions:

1. What kind of challenges did teachers have to face during the lockdown and post-lockdown emergency period in carrying out assessment processes?
2. How did they deal with these challenges?
3. What are the effects of the experience of distance teaching in terms of the evolution of the teachers’ praxeologies related to the task of realizing assessment processes?

The first two questions are aimed at characterizing teachers’ praxeologies, by highlighting the challenges they had to face to carry out the task of effectively developing assessment practices (research question 1) and the techniques adopted by teachers to face these challenges (research question 2). The third question is aimed at characterizing the evolution of these praxeologies, by highlighting the different ways in which internalization processes occurred (or did not occur), and the possible underlying reasons.

## Research methods

A fundamental aim of our research was to describe specific phenomena (the lockdown and post-lockdown teaching–learning experience) through the words of some of their protagonists (the teachers). Therefore, we conducted an international study carrying out semi-structured interviews with teachers at different school levels, which addressed the role of assessment within the mathematics classroom during the lockdown and post-lockdown periods.

### Research instruments for the data analysis: the IPA approach

Since we explored the phenomena under investigation in the same moment or shortly after they happened, we identified interpretative phenomenological analysis (IPA) as an effective tool to support our data analysis. IPA has the goal of analysing in detail how the people involved in specific phenomena perceive and make sense of them (Smith & Osborn, 2003).

As stressed by Smith and Osborn (2003), the IPA approach has the aim of understanding the complexity of meanings rather than measuring their frequency. An effective way to collect data for an IPA study is, therefore, to conduct semi-structured interviews, since it allows the researcher to engage in a dialogue with the participants of the study, modifying questions according to participants’ responses, with the aim of probing interesting ideas that arise.

IPA researchers adopt an iterative approach during the data analysis, moving back and forth through the data available in order to enter into their meanings and to grasp different perspectives (Smith et al., 2009). Moreover, IPA follows an idiographic approach to analysis, beginning with particular examples and slowly deducing more general claims or categories (Smith & Osborn, 2003).

Our analysis was articulated according to the following four stages, which characterize the IPA approach (Smith & Osborn, 2003):

1. *Looking for themes in the first case*: a transcript (the first case) is read and reread a number of times with the aim of annotating interesting ideas and identifying emerging themes corresponding to these ideas.
2. *Connecting the themes*: the themes, listed in chronological order after step 1, are reordered in a more analytical and theoretical way with the aim of making sense of the connections between emerging themes. This approach enables some themes to be clustered together and the identification of themes that emerge as superordinate concepts.
3. *Continuing the analysis with other cases*: during this phase, in which the analysis moves on to incorporate other cases, the aim is to discern repeating patterns and, at the same time, to acknowledge possible new emerging issues. The result of this step of the analysis is a final table of superordinate themes, selected with the aim of illuminating aspects of the topic under discussion.
4. *Writing up*: this final phase is aimed at translating the themes into a narrative argument “interspersed with verbatim extracts from the transcripts to support the case.” (Smith & Osborn, 2003, p. 76).

### Participants and research tools

In tune with the IPA approach, we first designed and implemented a series of semi-structured interviews with a small group of teachers. In total, we interviewed 48 teachers, equally distributed within the four countries and among the different school levels, from primary school (grades 1–4 or 1–5) to the last years of upper secondary school (grade 12 or grade 13, according to the different countries).

**Fig. 1:**
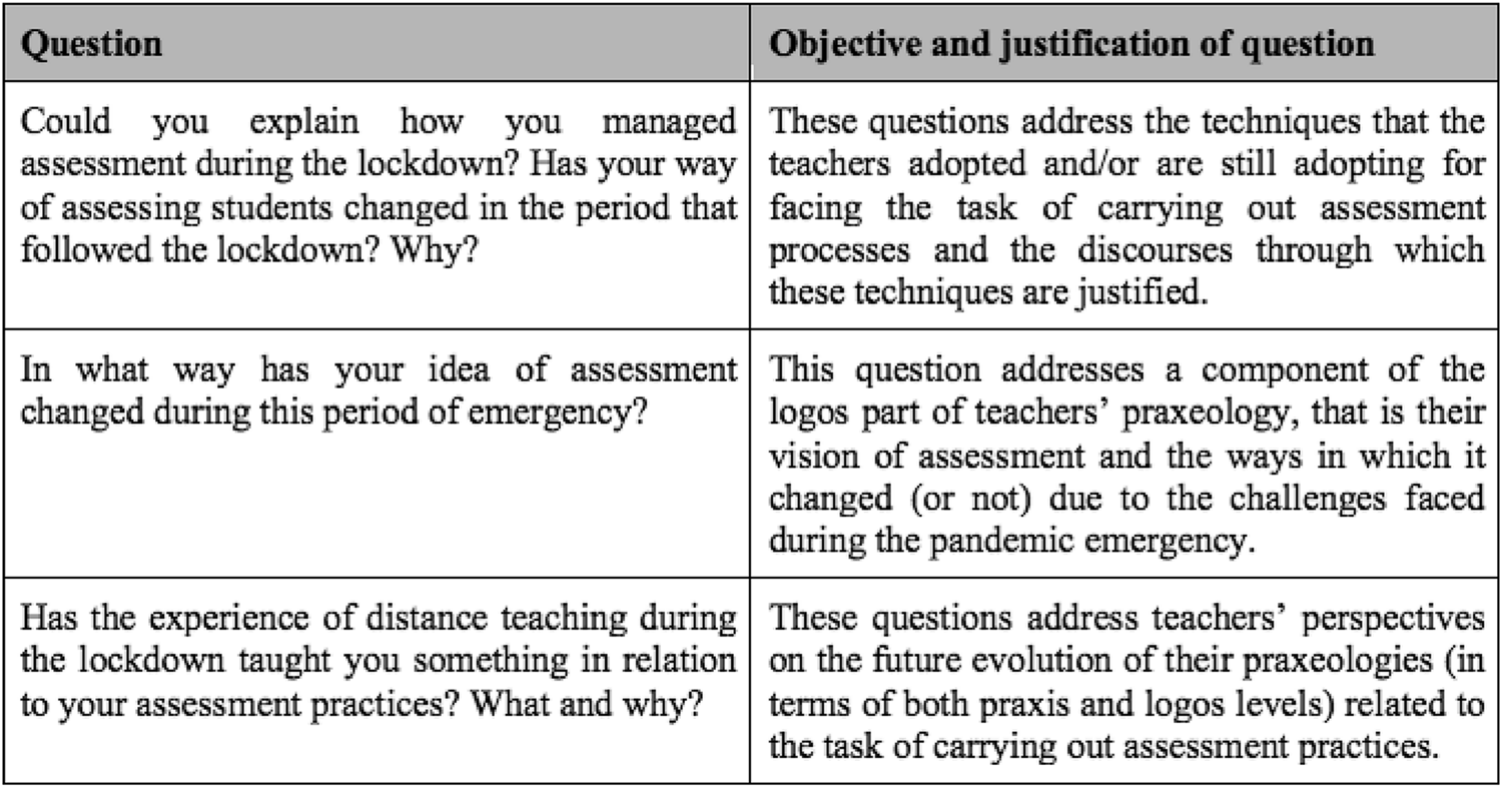
The three groups of central questions focused on teachers’ assessment practices

The interviewed teachers were enrolled on a voluntary basis among those who responded to the questionnaire presented by Aldon et al. (2021). Each interview lasted from 45 to 120 min. Researchers agreed, before the interviews, about the questions to be asked and their order, then the questions were translated into the interviewees’ languages. Each interview started with introductory aspects of teaching and learning during the lockdown. The questions within this first part of the interviews addressed general aspects of managing distance teaching to support students’ learning through specific methodologies, with the aim of highlighting the main changes that resulted from the teaching experience during the lockdown. The second part of the interview, which represents the focus of this paper, addressed aspects of assessment during the lockdown and post-lockdown periods. The teachers were asked to describe in detail their challenges when carrying out assessment and to reflect on how their ideas as well as their assessment strategies changed. Figure 1 shows the three groups of central questions belonging to this phase of the interview, with a description of the objective of each question.

### Exemplification of the data-analysis

The following exemplification gives detailed insights into the process of data analysis, developed according to the IPA approach. Since the results’ section presents the products and outline of themes reconstructed (step 4), the aim of this section is to make the analytical process explicit.

First, the four researchers individually developed the first phase of the IPA approach, by reading the interview transcripts, annotating interesting ideas in relation to assessment and emerging themes related to these ideas. Moreover, excerpts that were potentially interesting for the study were identified and carefully translated into the English language to be shared among members of the research group. A shared platform was established in order to collect and to cluster the different emerging themes, and various common (virtual) meetings were held to share initial connections among themes. At the end of this phase, three main clusters of themes were identified, each of them corresponding to one of the research questions.

The following excerpts from the data show examples of the first two main clusters.

Excerpt exemplifying cluster 1: “Since students are covered up to here (the nose), and they have a hat on their heads, the only thing we can see are the eyes, the feeling is of not getting anywhere, of not having the feedback and of being transparent, of not knowing where you are in the communication.” (Italian teacher, LS^1^)

Excerpt exemplifying cluster 2: “I had to change my ways of teaching and evaluating. I found two great apps on the internet that helped me give students problems in geometry and follow through on their solution. The software gives clues to the solution, and the students solve the problems using these clues. The system gives me online feedback. This allowed me to give more problems in geometry and follow the understanding of the students.” (Israeli teacher, US)

Both excerpts are highly relevant with respect to both the underlying theoretical framework and the research questions formulated above. The first excerpt addresses a challenge met by an Italian teacher, who focuses on the difficulty of activating a fundamental formative assessment strategy, that is, engineering effective classroom discussions as a basis of having evidence of student understanding. In the second excerpt, an Israeli teacher proposes her way of trying to face this kind of challenge, by describing the potential of a specific digital tool in supporting the activation of key formative assessment processes, such as monitoring students’ learning processes and supporting their work by means of specific feedback.

**Fig. 2:**
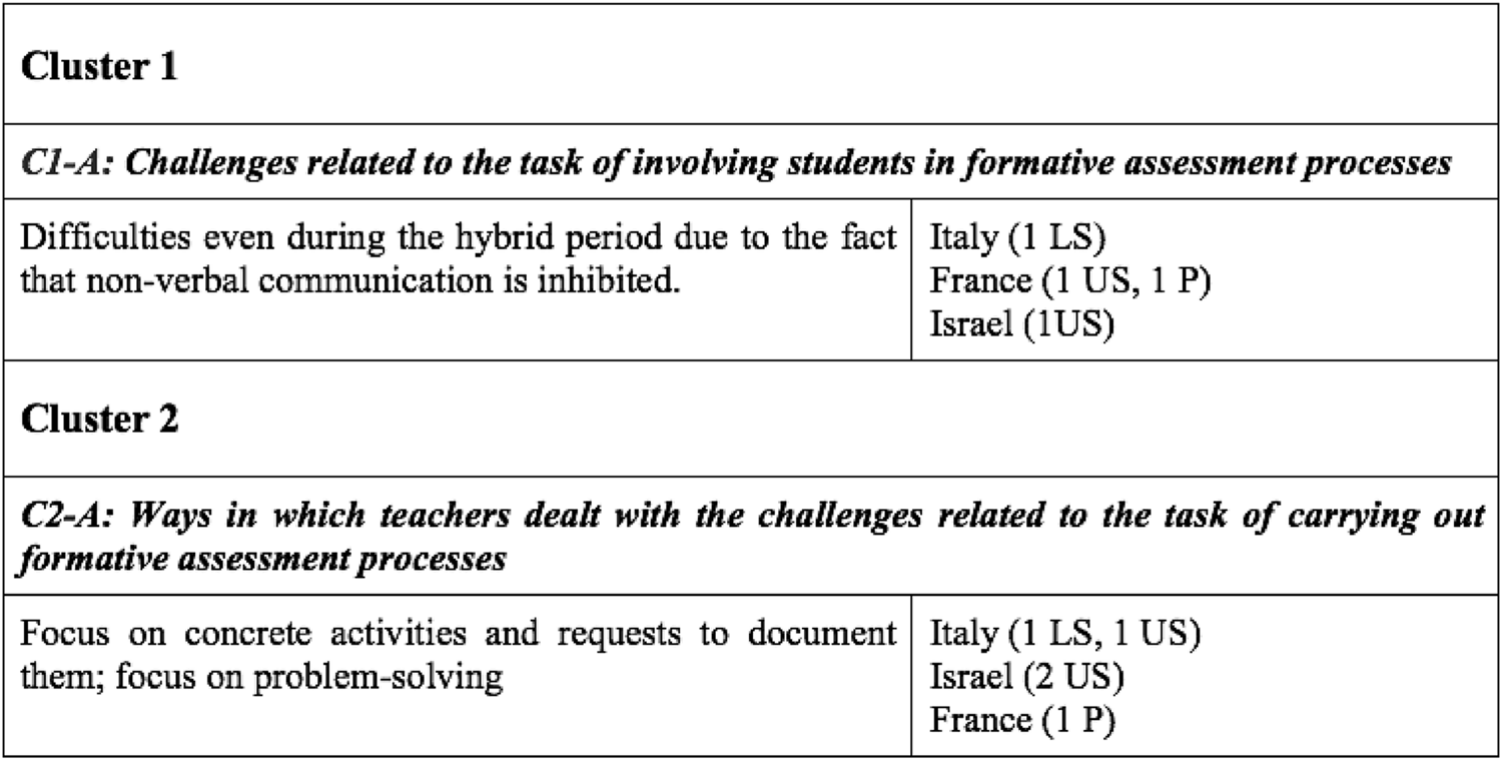
Fragment of the table used in the data analysis

After this first collection of data and the identification of three main clusters, the second and third phases of the IPA approach were developed, initially separately by the four researchers. The data were analysed systematically with the aim of reconstructing connections among the emerging themes and the collected excerpts, by identifying sets of sub-clusters for each of the three main clusters. The results of this analytical and clustering process were compared, both by using annotations within a shared document, and by holding various data-analysis meetings. Within these meetings, consensus was reached in respect to open questions related to a shared identification of sub-clusters that were relevant in relation to the research questions. In tune with the IPA approach, the researchers were interested in common as well as different country-specific findings, but the aim was not to develop a comparative study. At the end of this process, a set of sub-clusters was identified for each of the three main clusters and these sub-clusters were ordered systematically. On that basis, a table was constructed in order to produce a coherent and summarizing collection of the results of the data analysis. Figure 2 shows some lines in this table, in order to highlight how it is structured, according to clusters (lines 1 and 4), corresponding sub-clusters (lines 2 and 5) and descriptions of emerging themes within each sub-cluster (lines 3 and 6), together with the number of excerpts in the data of each country (column 2).

The two excerpts presented above exemplify data identified within the sub-clusters C1-A and C2-A in Fig. 2. The two sub-clusters give insights into the identification of a coherent and theory-based structure of the table of themes: not only are the main clusters 1 and 2 complementary, but also the sub-clusters have coherent and clear structure. In this part of the analysis, the data were treated phenomenologically, hence a systematic and theory-based analysis of the identified phenomena was carried out, whereas the excerpt was abstracted from the close contextual frame (e.g., in terms of the specific country or type of school). This means that the two excerpts presented above describe substantial phenomena, which relate to each other in terms of the research questions and the theoretical framework, even though they differ in terms of nationality and type of school.

During the third phase of incorporating all the data involved, repeating patterns were identified and a final table of clusters and sub-clusters was developed. Some excerpts were either re-assigned to other (sub-)clusters (e.g., because of the specific focus), some sub-clusters were merged (e.g., due to similar foci) and some sub-clusters were erased (e.g., not representing repeating patterns or relevant themes in relation to the research questions). The examples above are part of the final table of superordinate themes. The final step of describing the clusters and sub-clusters in detail gives a concise and theory-based description of the identified phenomena (see the next section).

## Results

In the following sections, we present the main themes that emerged from the analysis of the transcripts of teachers’ interviews. We structure this section into three main parts, each of them corresponding to one of the three research questions and to one of the main clusters we have identified.

Before presenting our analysis, it is important to take into account that, even if the four countries are close in relation to the general values their educational systems share, they differ from each other, for example in relation to the institutional framework according to which assessment techniques are justified. Therefore, teachers’ praxeologies, techniques and justifications of the techniques have to be interpreted and analysed with a strong reference to the institutional contexts. This explains why, in some cases, almost ‘opposite’ ideas appear within the same sub-cluster.

### The challenges faced by teachers in relation to the task of carrying out assessment processes

In this section, which addresses research question 1, we present the themes that emerged from the analysis of teachers’ interviews in relation to the first main cluster we identified. Our analysis of teachers’ interviews enabled us to identify three main sub-clusters, as follows: (1A) challenges related to the task of involving students in formative assessment processes; (1B) challenges related to the task of involving students in summative assessment processes; and (1C) more general challenges associated with the realization of both summative and formative assessment processes.

As regards *sub-cluster 1A*, one of the main challenges that teachers faced during the lockdown period was related to different technical problems that prevented them from effectively activating typical formative assessment strategies, such as designing and conducting whole classroom discussions aimed at eliciting evidence of students’ understanding. Teachers related these issues to different factors, including the following: the initial lack of experience for both teachers and students in the use of specific digital tools (especially communication tools) and the corresponding need to identify ways of using these new tools to activate known assessment techniques; a lack of clear institutional guidelines, especially at the beginning of the lockdown period, about possible ways of managing distance teaching (in most of the countries, each school had to organize distance teaching autonomously); and students’ lack of the minimum tools to participate in online interactive lessons (especially in the case of students in disadvantaged economic conditions). Teachers often stressed the influence of these technical problems on their capability to realize formative assessment processes, as in this excerpt:

“In some cases, especially at the beginning, we had only four, five students with the camera on, a dozen who said that the microphone did not work... Anyway, I didn’t have the possibility to mediate a distance assessment through discussions, I missed that part of the assessment. So, in some cases, the assessment was penalizing students.” (Italian teacher, LS)

Some teachers reported that, during the first post-lockdown period, these technical difficulties even increased, due to the need to find tools to support communication with the students. This happened in particular in the case of countries (such as Italy and France) where, especially at the upper secondary school level, schools had to organize teaching in order to enable half of the students to follow lessons at a distance.

Another challenge belonging to *sub-cluster 1A* is related to specific difficulties faced by teachers in following students’ processes due to the impossibility of activating multimodal communication, by looking, for example, at students’ gazes or at their gestures. In particular, teachers complained that a lack of multimodal communication prevented them from effectively investigating “where their students are in their learning process”:

“In teaching situations where the students are present, I look at the students’ faces a lot. If I explain something and the three high achieving students look confused, I know: This was not the best way. Something like this I hardly recognize in video conferences.” (German teacher, US)

“I was never satisfied because I didn’t have my finger on the pulse anyway, I didn’t feel them (the students) ... I didn’t feel the chemistry I usually perceive in the class.” (Italian teacher, P)

“…It is quite difficult to conduct a lesson without seeing the students’ gestures, their facial expressions; you cannot know whether they understand you or not.” (Israeli teacher, US)

In tune with these ideas, some teachers expressed their feelings about the phenomena lived during the lockdown period, using the term “a brake on interaction” to describe their experience with distance teaching.

The difficulties met in monitoring low achievers’ learning processes and in supporting them individually by means of adaptive forms of support represent the third type of challenge belonging to *sub-cluster 1A*. These difficulties, reported by teachers from all the four countries, are testified to in the following excerpt:

“I met them once a week for an hour in small groups of 10. But it was only talking about what they hadn’t understood. So, I cannot say that I followed them individually; I didn’t know how far they were in their learning...” (Italian teacher, P)

Our analysis highlighted two main challenges reported by teachers in relation to students’ involvement in summative assessment processes (*sub-cluster 1B*).

The first challenge was mentioned by teachers from all the countries, and it is related to their need to ensure the reliability of the examinations and of being sure that students did the examinations by themselves, without external help. The main concern of these teachers was how to prevent cheating during the summative assessment, as shown in the following excerpts.

“As for reliability, I do not know, it is complicated to trust them, whether they cheat or do not cheat. And that they do not want to run the camera on a test. This makes the assessment challenging.” (Israeli teacher, US)

“To be sure that it was the student doing the assessments because when you are at a distance you don’t know who is doing what, even with LaboMep (a popular software in France), you don’t know who is behind the computer.” (French teacher, US)

The following excerpt shows that this challenge continued to accompany the teachers during the first post-lockdown period, during which, in some countries, some students were in their homes while other students were in their classrooms.

“Now I propose written tests when students are present, and so I have to prepare two tests. So, I have to propose the tests on two consecutive days during the week to ensure that all the students can do the test when they are present. And so, it gets complicated...” (Italian teacher, US)

The second main challenge belonging to *sub-cluster 1B* is related to teachers’ difficulties in identifying the right objects of assessment during the lockdown period. In the four countries, the teachers, especially those whose students were involved in national final examinations, expressed their worries due to the lack of clarity about the mathematical topics involved in the final examinations and about the ways of assessing them (in Italy, for example, the Ministry took the decision to change the structure of the final examination and these changes were communicated at the last moment). These worries are made explicit in the following excerpts:

“Obviously students in the fifth year (grade 13) are worried about the final written examination because they have to do it anyway and, therefore, they want to know what will happen.” (Italian teacher, US)

“The institute asked us not to emphasize the issue of the assessment. We do not know whether the Bagrut examination will be held or not; this affects the assessment process. Despite saying that the Bagrut exams perhaps will not be conducted, they can change their mind. In this case, we cannot anticipate which topic will be tested. In short, a big mess.” (Israeli teacher, US)

Teachers also reported more general challenges related to both formative and summative assessment (*sub-cluster 1C*). Due to space limitations, we mention, in particular, two challenges reported by teachers of the four countries, which highlight the key role played by the teachers’ interactions with other important protagonists of assessment processes (besides students):

- difficulties in collaborating, for the co-design of assessment tasks or strategies, with colleagues who approach assessment with different perspectives;
- difficulties related to parents’ interference during the synchronous activities and during tests.

### The ways in which teachers dealt with the challenges related to the task of carrying out assessment processes

This section addresses research question 2. In line with the results presented in the previous section, we focus on two main sub-clusters that can be identified within the second cluster. *Sub-cluster 2A* relates to the challenges in involving students in formative assessment processes, while *sub-cluster 2B* relates to the challenges in involving students in summative assessment processes.

In relation to *sub-cluster 2A*, one of the ideas mentioned by teachers was creating a relationship of trust with both students and their families, with the aim of fostering their authentic involvement in formative assessment processes, activating them as the owners of their own learning. In some cases, for example, teachers reported on conversations with students that they carried out systematically, aiming at consolidating their relationship with them. Some teachers explicitly mentioned that they bypassed tests and “preferred (cultivating) relationships” (Italian teacher, LS). This theme is mentioned only by groups of French and Italian teachers. It seems that the reason could be related to the teaching–learning organization in the different countries and to specific institutional guidelines given by the ministry of education or by the school principals, such as in the experience reported by this teacher:

“...the only direction that we had when we saw that it lasted a little longer, was that each teacher had to have contact with his/her students, a remote contact...” (French teacher, US).

The second theme belonging to *sub-cluster 2A* is related to the idea of focusing on concrete activities and on problem-solving as a way of engineering learning tasks that elicit evidence of student understanding. The teachers from all four countries reported on “making the students build things” (Italian teacher, LS), initiating problem-solving activities, “explaining with hands, with a small diagram” (French teacher, LS) or using apps that helped to support students’ exploration of specific problems (e.g., in geometry). These descriptions both hint at the use of multiple representations (iconic, symbolic) and materials for involving students in formative assessment processes, as well as to the reflection of tasks suitable for formative assessment.

This strategy was often combined with a focus on collaborative ways of working, since fostering group work in breakout sessions for collaborative learning seemed to be an effective way of stimulating students to become resources for their classmates.

The third theme belonging to *sub-cluster 2A* is related to the challenge of monitoring students’ processes, that is, of investigating where students are in their learning. Since collecting and checking students’ written work (e.g., homework) were found to be time consuming for teachers, most of them declared that they adopted different approaches to face this challenge. The approaches that were mainly mentioned by the interviewed teachers were as follows:

- focusing on oral communication with groups of students or initiating collective discussions;
- focusing on group-centred online diagnostics, e.g., the use of polls to have a quick overview on students’ understanding and participation, as stressed by this teacher: “to check first: ‘Yes, 3 out of 25 know it. Or is it 17 out of 25’. If the latter is the case, then I can continue.” (German teacher, US);
- using ordinary summative tests without giving marks (this choice was also related to teachers’ assessment routines and to the perceived lack of reliability of online tests).

The fourth theme we identified in relation to the challenge of involving students in formative assessment processes (*sub-cluster 2A*) refers to the concept of feedback. The data showed that the interviewed teachers were aware of the importance of written and oral feedback as a tool both to enable students to reflect on their learning and to foster their motivation. In respect to written feedback, although some teachers stressed the difficulty “to write and to evaluate the students … as a maths teacher” (French teacher, US), they also reported on new approaches—especially in terms of motivation and valuing the students, seen as a central aspect for the mathematics classroom:

“I think that due to the distance situation they got feedback that motivated them. Because, we as teachers tend to concentrate on the mistakes (…). So just value something.” (German teacher, US).

In terms of oral feedback, teachers from all countries described similar situations concerning synchronous moments of one-to-one (or small groups) communication as consulting time for the students. Teachers reported on the activities of calling students in the morning to give them the programme for the day and calling them back in the afternoon to “check what they have done. And then I called my special needs students, four, five of them, and then we worked by phone as well” (French teacher, P).

We identified two main themes related to the ways of dealing with the challenge of involving students in summative assessment processes (*sub-cluster 2B*).

The first theme refers to the challenge of not knowing if the assessment results were reliable or not during distance teaching. Some teachers reported that, in order to face this challenge, they designed ad hoc tasks to be able to understand if students had used specific software to do written tests at a distance:

“I deliberately set some exercises that Photomath solved in an absurd way to find out the students who had used this app.” (Italian teacher, US).

Other teachers declared that they combined written and oral tests in order to check if students had really done the written tests by themselves or if they were helped.

In some cases, teachers reported on the ways in which this kind of challenge was addressed even during the post-lockdown period, in those schools in which some students alternatively had to follow lessons at distance. In these cases, teachers organized lessons in order to make all the students undertake the written tests when they were at school.

Many teachers also discussed the tasks for checking the students’ real understanding, especially by asking students to share the whole reasoning process (not only the final product), by developing argumentative processes concerning their solutions of tasks. Some teachers especially highlighted the role of open-ended assignments as being valuable within distant-learning situations:

“In some way, you can also do this online: you can give open-ended assignments, to be commented on and, in this way, you can see if they understand or not.” (Italian teacher, LS)

The second theme belonging to *sub-cluster 2B* is related to the object of assessment. Indeed, many teachers reported a question that most of them asked themselves during the distance teaching period, namely, what should be assessed? These teachers stressed the fact that, during distance teaching, the focus of their assessment changed completely, since they realized that, instead of assessing students’ performance, it was necessary to assess only what was really observable (e.g., participation, commitment, resilience…) and to take into account all the information that teachers already had about their students.

In this context, some teachers especially highlighted the change in their views on assessment in terms of giving respect to dimensions not closely related to mathematical performance, as the following excerpts highlight:

“During the pandemic I came to know that assessment is more than an exam, we can evaluate the students. We can evaluate the students through their work: students’ rigour; their participation; their seriousness.” (Israeli teacher, LS)

“...Assessing critical analysis, concrete participation, commitment and punctuality in tasks, commitment and punctuality during the online meetings, because this is also part of a 360-degree assessment.” (Italian teacher, LS)

### Effects of the experience of distance teaching in terms of the evolution of the teachers’ praxeologies related to the task of realizing assessment processes

In this section, which addresses research question 3, we present the themes that emerged from the analysis of teachers’ interviews in relation to the third main cluster we identified. During the interviews, in particular when answering to the third group of questions within Fig. 1, teachers described an ongoing evolution of their praxeologies. In some cases, this evolution was characterized by the internalization of components that were completely external to the teachers’ praxeologies and became internal as a result of their reflections on the new practices developed during the lockdown period. In other cases, distance teaching fostered the completion of an internalization process that had started before the COVID-19 crisis itself, or that only contributed to the consolidation of already existing praxeologies. Sometimes, the internalization process did not happen, due to constraints that prevented the transformation of external components into internal ones; for example, some teachers perceived the lockdown period as a ‘blank period’, characterized by experiences that were too far from the reality of the ‘ordinary’ classroom to be internalized.

In light of these observations, in order to characterize the complexity of the phenomena of internalization emerging from the teachers’ reflections on the effects of the distance teaching experience on their praxeologies, we categorized these reflections according to the following:

- the typologies of components that have been internalized: components belonging to the praxis level of teachers’ praxeologies (P); components belonging to the logos level (L);
- the levels of internalization of these components: consolidation of pre-existing components of teachers’ praxeologies (a); internalization of new components (b); lack of internalization (c).

From this categorization, six specific sub-clusters could be identified, each of them corresponding to one combination of categories (P) and (L) with categories (a), (b) and (c). This categorization is the result, not of a direct observation of what teachers actually did before and are now doing in their assessment practices, but of an investigation of the teachers’ reflections, with the aim of making their perspectives on the effects of the distance teaching experience explicit.

If we focus on *sub-clusters P-a* and *P-b* (consolidation of pre-existing components and internalization of new components within the *praxis level* of teachers’ praxeologies), we can observe that, in their interviews, teachers mainly refered to techniques (new or pre-existing ones) related to a formative conception of assessment. These techniques often involved the use of digital platforms or specific digital tools to realize different processes, such as the following:

- collecting students’ written protocols to better monitor students’ learning processes (e.g., with Google Classroom) and organizing classroom discussions starting from students’ answers;
- boosting students’ sharing of materials with the teacher and classmates and the digital correction of students’ shared materials (e.g., with graphic boards) to provide continuous feedback to students;
- giving students the opportunity to compare their answers with those of their classmates (e.g., with Padlet) and to become resources for them, realizing peer assessment processes;
- fostering cooperative learning, by organizing virtual meetings between students (e.g., with Google Meet or Zoom);
- designing digital tests that provide immediate feedback to students (e.g., by Google Form), in order to support them in self-assessment processes.

The following excerpts are aimed at exemplifying the typical teachers’ reflections that belong to *sub-cluster P-a* (the first excerpt) and to *sub-cluster P-b* (the second excerpt).

“So, more and more, but that’s an evolution that I’ve had in the last few years, based on discussions with colleagues, I’m assessing over time [...] More and more I watch, assess and take notes when they work. [...] then we continue to work on it, and then if there are [still difficulties] I take a small group with me.” (French teacher, P)

“...Last year (during the lockdown), I used to look at their protocols, to fix them, to organize them logically, to design a presentation, and then, the next day, to discuss with students starting from their work. This is absolutely a novelty of this very fast digitization that happened in recent months.” (Italian teacher, LS)

Some teachers (especially lower and upper secondary teachers) declared that they deliberately decided to stop using some of the techniques adopted during the distance teaching period, such as meeting small groups of students within digital platforms outside the lessons or collecting students’ materials in shared folders and correcting them. Through their reflections, which belong to *sub-cluster 1-c*, teachers justified this lack of internalization of specific techniques by referring to the fact that they were found to be too demanding for teachers, as the following excerpt indicates:

“Assessment during the COVID-19 era was difficult and needed a lot of time to do it. […] There are many methods to assess your students, but these methods need time.” (Israeli teacher, US)

If we focus on the characterization of the *logos level* of teachers’ praxeologies related to assessment, we can first observe that no teachers described a lack of internalization of components belonging to the logos level of praxeologies (*sub-cluster L-c*). Most of the teachers’ reflections, instead, belong to *sub-cluster L-a*, since, often, the process that teachers described is mainly that of consolidation of pre-existing ideas about assessment that were already components of the logos level of their praxeologies.

In some cases, teachers testified to have *consolidated a summative conception of assessment*, focused on the need to find strategies useful for preserving what they considered to be an objective way of assessing. These teachers (mainly upper secondary teachers) declared that when they came back to school after the distance teaching period, they also came back to their previous ‘more objective’ ways of assessing students, as illustrated by the following excerpt:

“Last year I changed my approach enough to give marks at the end of the year. Instead, this year I have tried as much as possible to return to a fairly traditional approach that consists in collecting marks from written tests.” (Italian teacher, US)

These teachers often also refered to institutional constraints, such as the need to collect a certain number of marks by the end of the term, or of preparing students for final examinations. In some cases, teachers explicitly declared they did not trust students’ opinions about their assessment, as the following excerpt shows:

“…We need to prepare students for the Bagrut exams. This is my measure of success as a teacher as well. Honestly, I do not trust the students’ opinion to give me a credible assessment of their academic achievement...” (Israeli teacher, US)

In other cases, teachers spoke about distance teaching as an opportunity to *consolidate a formative vision of assessment*, since this experience enabled them to verify the effectiveness of many of the techniques that they were used to applying before the lockdown period, and to re-discover the importance of involving all the actors (the teacher, the students, their peers) within the assessment process, by means of peer- and self-assessment practices. According to these teachers, assessment should not be identified with ‘measuring students’ performance’ or with ‘attributing scores to students’. Instead, it should be aimed at ‘narrating the story of the students’, with the aim, on one hand, of supporting the teachers in adapting their teaching, and, on the other hand, of enabling students to become aware of their learning, as the following excerpt testifies:

“In my view, assessment should have two roles. It is a tool for teachers to follow the students’ progress and to adapt their instruction, and for students it is a tool to help them evaluate their actual learning...” (Israeli teacher, P)

Some of the teachers who manifested a formative vision of assessment described a process of internalization of specific ideas about assessment as new components within the logos level of their praxeologies (*sub-cluster L-b*). The main novel ideas mentioned by teachers were as follows:

- the importance of focusing on students’ emotions during the assessment process, referring to formative assessment practices as effective tools to prevent the negative emotions usually triggered by summative tests;
- the importance of actively engaging parents within formative assessment processes (especially in primary school) in order better to coordinate the pupils’ experiences out of school with those in school;
- the need to coordinate assessment practices at different levels (the classroom level, the school level, the level of national assessment) and to focus assessment more on the mathematics curricula than on the textbooks.

## Final discussion

In this paper, we present the main results of a study aimed at investigating the impact of the COVID-19 crisis on teachers’ assessment practices, and the teachers’ reflections on such practices. The study delineates teachers’ perspectives on the *past* (the lockdown period) and *present* (the post-lockdown period) of their own didactical experiences, in terms of assessment practices. In line with related recent studies (e.g., Drijvers et al., 2021), our results show that assessment was a big challenge for teachers in the distance teaching situation. By analyzing teachers’ practices and their reflections some months after the novelty of synchronous distance teaching, this study addresses recommendations suggested by first moment studies (Aldon et al., 2021; Drijvers et al., 2021).

The analysis carried out in this paper also focused on the identification of relevant themes that emerged from teachers’ reflections at the meta-didactical level, about the ways in which the lockdown and post-lockdown emergency periods triggered (or did not trigger) an evolution of their assessment practices, helping us to delineate possible scenarios about the *future* of these practices. These findings indicate that teachers see a need of assessing what should really be valued rather than “merely assessing what is relatively easy to assess” (Bakker et al., 2021, p. 18).

Each of the main three clusters of themes presented in the previous section represent the answer to one of the research questions that guided this study. The themes within cluster 1, highlighting the main challenges faced by the interviewed teachers in carrying out assessment processes (research question 1), enabled us to identify the tasks that characterized the teachers’ praxeologies that are objects of their reflections (in line with related studies such as that of Nilsberth et al., 2021). The themes within cluster 2, stressing the ways in which the interviewed teachers faced the challenges they met (research question 2), enabled us to highlight the techniques that characterized teachers’ praxeologies during the lockdown and post-lockdown emergency periods. Finally, the themes within cluster 3 suggested the formulation of specific hypotheses about the long-term effects of the experience of distance teaching on teachers’ praxeologies related to the task of realizing assessment processes (research question 3).

As we stressed before, the aim of this study was not to infer general conclusions from the analysis of the data we collected, but to delineate a picture of how the challenging experience of carrying out assessment processes in mathematics during the lockdown and post-lockdown emergency periods were interpreted by specific individuals, affecting, in different ways, their praxeologies.

What can be stated with certainty is that this experience enabled some teachers to discover other ‘possibilities’, that is, other possible ways of developing assessment processes, potentially enlarging their repertoire of assessment techniques. This is in tune with the results of other studies, such as the one by Fitzmaurice and Fhloinn (2021), who highlighted that lecturers welcomed the changes that technology brought to their teaching.

Moreover, at the same time, this experience also gave many teachers the opportunity to highlight the value of formative assessment practices and to develop or consolidate, in this way, a formative vision of assessment. Two main reflections developed in relation to this result.

The first reflection refers to the ‘stability’ of the changes and transformations of assessment practices declared by teachers. What we presented in the previous section, in fact, is the result of the analysis not of the actual evolution of teachers’ didactical praxeologies, but of the teachers’ interpretation of this evolution. Will these declared changes be permanent or only transitory? What we can hypothesize is that if the processes described by teachers correspond to a real internalization of stable components within the praxis and logos level of their praxeologies, they will also refer to these new internalized techniques and corresponding justifying discourses when they develop their future practices. If this internalization is not real, teachers will probably soon return to their previous approach to assessment.

The second reflection is related to the key role played by cultural, institutional and contextual influences, testified to by the differences in what was declared during the interviews between teachers from different countries (in tune with Drijvers et al., 2021), but also between teachers working at different school levels or in different school contexts.

This point leads to a further reflection on the characteristics of the phenomena of changes in teachers’ praxeologies described during the interviews. Could these phenomena, developed at a local level, engender more global phenomena? The need for a ‘globalization of local changes’, especially in the case in which these changes lead to the development of a more stable formative vision of assessment, is realized by the reflection of one of the teachers who participated in our study, who denounced the risks related to the perpetuation of an archaic perspective of school as ‘marks-maker’:

“...I would like that school becomes a place of authentic confrontation, of authentic sharing of ideas. This space must be freed from the anxiety of numbers… which is, in my opinion, a cage ... if school does not become like this, it is destined to disappear due to its irrelevance, that is, to disappear due to the inertia of things ...” (Italian teacher, LS)

The internalization processes described by teachers could be metaphorically represented as waves triggered at a local level. These waves will be able to spread only if political and educational institutions promote and support the stabilization of the changes at a more global level. In our opinion, this could happen by focusing on educational programmes aimed at deepening teachers’ professional development that could really support teachers in realizing authentic formative assessment practices.

## Footnotes

1 In the following, the acronyms LS, US and P are used, respectively, to indicate lower secondary school (grades 6–8 or 6–9), upper secondary school (grades 9–12 or 9–13) and primary school (grades 1–4 or 1–5).
